# Decision-making preferences and risk factors regarding early adolescent pregnancy in Ghana: stakeholders’ and adolescents’ perspectives from a vignette-based qualitative study

**DOI:** 10.1186/s12978-020-00992-x

**Published:** 2020-09-11

**Authors:** Luchuo Engelbert Bain, Seda Muftugil-Yalcin, Mary Amoakoh-Coleman, Marjolein B. M. Zweekhorst, Renaud Becquet, Tjard de Cock Buning

**Affiliations:** 1 Athena Institute for Research on Innovation and Communication in Health and Life Sciences, Vrije Universiteit Amsterdam, Amsterdam, The Netherlands; 2 Infectious Diseases in Lower Income Countries (IDLIC) Team, Inserm, Bordeaux Population Health Research Centre, University of Bordeaux, Bordeaux, France; 3 Lincoln International Institute for Rural Health (LIIRH), College of Social Science, University of Lincoln, Brayford Pool, Lincoln, Lincolnshire UK; 4 Department of Epidemiology, Noguchi Memorial Institute for Medical Research, University of Ghana, Legon, Ghana

**Keywords:** Decision making, Adolescent, Pregnancy, Ghana, Preferences

## Abstract

**Background:**

Worldwide, over half of the adolescent pregnancies recorded are unintended. The decision to continue the pregnancy to term or to opt for an abortion is a constant dilemma that is directly or indirectly influenced by stakeholders and also by the wider social environment. This study aimed at understanding the perceived decision-making preferences and determinants of early adolescent pregnancy in the Jamestown area of Accra in Ghana.

**Methods:**

A vignette-based qualitative study design was used. Eight focus group discussions were carried among various purposively selected groups of participants: parents, teachers, adolescent students who had not been pregnant before, and adolescents who had had at least one pregnancy in the past. The vignette was a hypothetical case of a 15-year-old high school student who had not experienced her menses for the past 6 weeks. The data were analyzed using a thematic analysis approach.

**Results:**

Lack of parent-daughter communication, the taboo on discussing sex-related issues in households and weak financial autonomy were considered to be the main contributing factors to the high early adolescent pregnancy rates in the community. Partner readiness to assume responsibility for the girl and the baby was a key consideration in either continuing the pregnancy to term or opting for an abortion. The father was overwhelmingly considered to be the one to take the final decision regarding the pregnancy outcome. Irrespective of the fact that the respondents were very religious, opting for an abortion was considered acceptable under special circumstances, especially if the pregnant adolescent was doing well in school.

**Conclusion:**

Inadequate and inappropriate communication practices around sexuality issues, as well as weak financial autonomy are the major predictors of early adolescent pregnancy in this community. The father is perceived to be the main decision maker regarding a young adolescent’s pregnancy outcome. Policy-makers should carefully evaluate the implications of this overwhelming perceived desire for the father to be the final decision-maker regarding adolescent pregnancy outcomes in this community.

## Plain English Summary

The majority of pregnancies among adolescents are unintended. It is more difficult for adoelscents compared to elderly women to decide upon their pregnancy outcomes. With a dsiturbingly high number of adolescents getting pregnant relatively early in James Town, Accra, we carried out 8 focus group discussions using a 15 year old pregnant adolescent as a hypothetical case, to understand the decision making preferences of stakeholders and adolescents, as well as their perceived determinants of early adolescent pregnancy in this community. Poverty and lack of parent – adolescent communication on sexuality issues were the main identified factors that led to early adolescent pregnancy in this community. Despite being a very religious community, going in for an abortion was considered acceptable, especially if the adolescent had a good academic record. The readiness of the partner to assume responsibility over the pregnacy was the main determining factor in deciding either to keep the pregnancy to term, or to opt for an abortion. The father was considered by the majority of respondents to be the one to take the final decision regarding the pregnancy outcome of the adolescent.

## Background

Unintended pregnancies remain a serious public health concern worldwide [1]. Over 40% of recorded pregnancies are unintended, and over half of these pregnancies do terminate in abortions [1]. This burden is disproportionately higher amongst adolescents [1, 2]. In Ghana for instance, 69.4% of unintended pregnancies occur among adolescents [3]. Adolescent pregnancies have adverse health and socio-economic consequences for the adolescent, the baby, and the community [4–6]. These consequences include high rates of caesarian section, prolonged labor, early infant deaths, and low birth weight [5]. Early pregnancy can impede the adolescent from attaining her academic potential, leading to substantial economic consequences over her life course [6]. With the growing evidence of strong intergenerational occurrence of teenage pregnancy between a mother and daughter, coupled with its known adverse socioeconomic and health outcomes for the teenage mother and the baby, adolescent pregnacy prevention remains a top public health priority globally [7, 8]. Understanding context specific predictors of adolescent pregnancy, as well as the decision making process, are relevant in establishing appropriate preventive interventions, as well as to provide respectful counseling services for pregnant adolescents.

A case – control study from the Sunyani Municipality in Ghana identified place of residence, socioeconomic status, and the adolescent’s occupation as main determinants of adolescent pregnancy [9]. Findings from a qualitative study among teenage mothers in the Ga East Municipality in Accra reported transactional sex, sexual violence and exploitation, and adolescent’s desire to command respect from the society as main reasons why adolescents became pregnant [10]. Engaging in sexual activity was justifiable mainly for economic reasons by teenage mothers from the Bolgatanga and James Town (Accra) areas in Ghana [11, 12]. Pregnant adolescents, like other women with unintended pregnancy, are generally faced with the dilemma of either continuing the pregnancies to term, or going in for abortions. This is even more challenging for adolescents, generally due to lack of policy guidance to guide the procedure, as well as social and cultural considerations. The discussion of who should be involved in the decision-making process and who should take the final decision is usually complicated one. Understanding the community perceptions regarding the risk factors for adolescent pregnancies, as well as the community-perceived ideal decision-making process and actors, could be useful in framing culture-specific health care interventions, as well as friendly health care services for pregnant adolescents. Research focusing on early adolescent pregnancy predictors (< 15 years of age) and decision making preferences are sparse. This was a main justification to carry out this study.

**Fig. 1:**
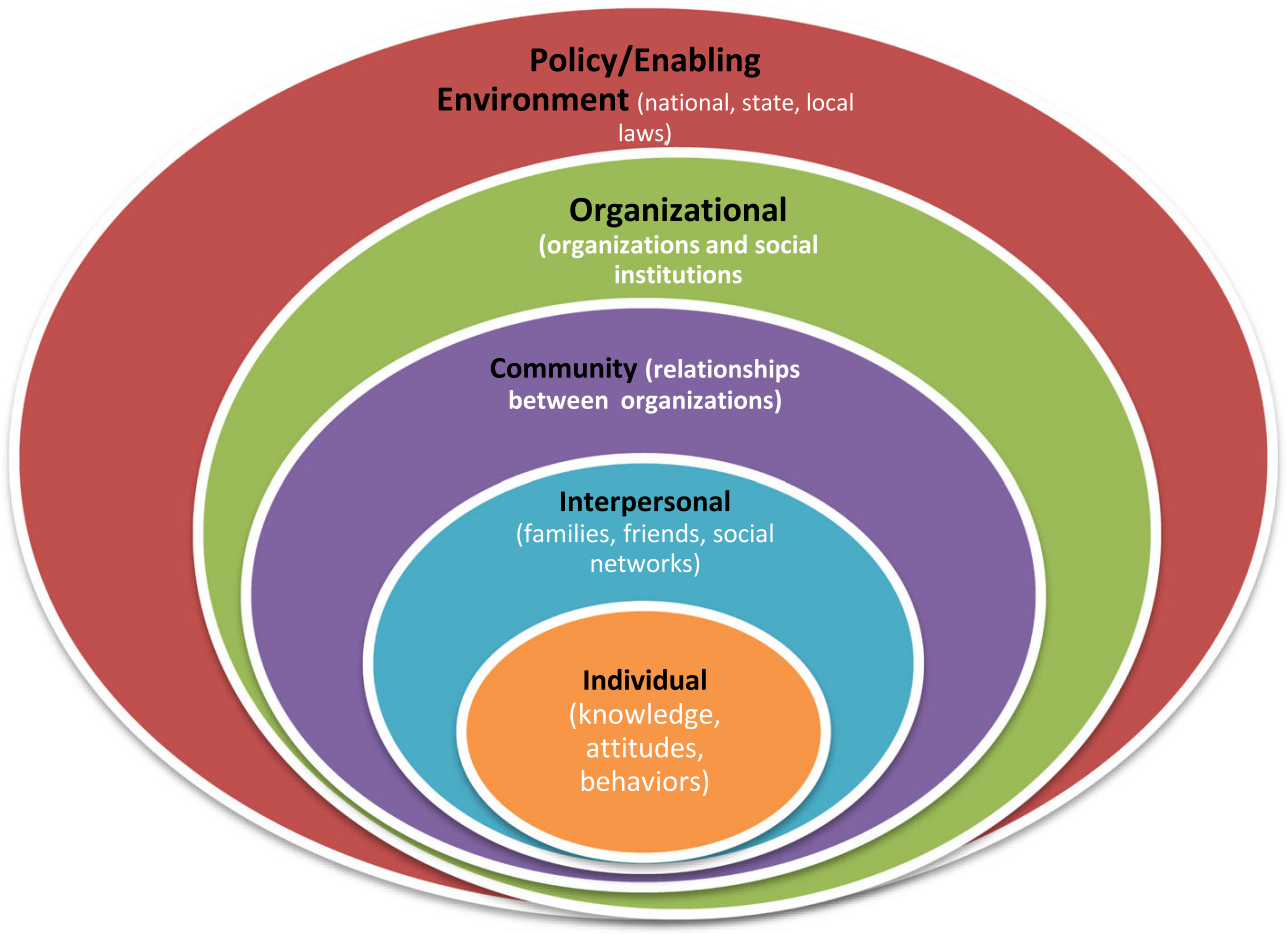
Hierarchical levels in the socioecological model [Adapted from the Centers for Disease Control and Prevention (CDC), The Social Ecological Model: A Framework for Prevention, http://www.cdc.gov/violenceprevention/overview/social-ecologicalmodel.html (retrieved June 21, 2019)]

The majority of adolescent pregnancy prevention programs are aimed at the individual and/or interpersonal levels of intervention. Such approaches are reductionist, as psychosocial (stigma), cultural, institutional, environmental, economic and health policy factors play significant roles in shaping, for instance, individual behavior and access to health care services [13]. Positing on the premise that early adolescent pregnancy and decision making is complex, the socioecological model was considered appropriate to allow for a more holistic understanding of the individual, interpersonal, organizational, community, or policy factors involved. The Social Ecological Model (SEM) [14, 15] posits that behavior and decision making are shaped by individual, relationship, community, and societal factors. It is a theory-based framework for understanding the multifaceted and interactive effects of personal and environmental factors that determine behaviors, and for identifying behavioral and organizational soft spots and intermediaries for health promotion within organizations. There are five nested, hierarchical levels of the SEM: Individual, interpersonal, community, organizational, and policy/enabling environment (Fig. 1).

## Methodology

### Study setting

Accra is the capital city of Ghana and has a predominantly young population. In 2012, 38.8% of Ghana’s population was under the age of 15 [16]. Jamestown is a small coastal town located in the heart of the Ashiedu Keteke Sub-metro in the capital of Accra. It’s one of the oldest suburbs. It is located among a cluster of coastal suburbs which are characteristically poor (low-income) and overpopulated. The area comprises a mix of traditional indigenous Ga people who form the largest sub-ethnic group (18.9%). Christians constitute the largest religious group (83.0%), followed by Muslims (10.2%). The indigenous people of the area are mainly engaged in fishing activities, while petty trading, hawking and head porting (kayayee) are the mainstay of the migrant population [16].

### Study design

A vignette-based focus group discussion design was adopted to investigate the risk factors of early adolescent pregnancies (≤15 years old). Vignettes use short stories about hypothetical characters in specific circumstances, and the interviewee is invited to respond to that situation [17, 18]. They enable participants to present their understanding of the topic in their own terms, allowing for actions to be clearly explored in context; people’s judgements are clarified without getting personal, and thus sensitive topics can be better understood. We hypothesized that deciding on an abortion is one of the options in this case. Research suggests that responses from vignettes generally mirror the social reality of the area under study [17, 18]. In this study, we sought to ascertain how various groups (never pregnant adolescents, ever pregnant adolescents, parents, and teachers) would react to a hypothetical 15-year-old adolescent who is 6 weeks pregnant, the possible causes of this girl finding herself in this situation, how this situation could have been prevented, and the perceived role players in decision making, either to allow the pregnancy to go to term or to choose an abortion.

### Research participants

To obtain a holistic view to answer our research question, we carried out eight focus group discussions (FGDs) (54 participants in total) among four groups of purposively selected research participants: parents (2 FGDs) teachers (2 FGDs) adolescents who had never been pregnant (2 FGDs), and adolescents who had been pregnant at least once (2 FGDs). Adolescent mothers were included to investigate the role of a previous pregnancy experience on perceptions towards future pregnancy risk and decision-making preferences. Gender equity was sought as much as possible in the constitution of the teacher and parent groups. To ascertain the influence of a pregnancy experience on the perceived decision making, we decided to include adolescents who had been pregnant before. Adolescents were recruited from the adolescent health unit of the Ussher Polyclinic in Jamestown, Accra. This is the main public health facility that serves the population in this area. Teachers were purposively selected from two schools in the area (Sacred Heart Technical Institute Secondary school and the Bishop Girls’ primary school). Parents attending the outpatient department of the clinic were approached regarding the research. After attaining the required goal of eight participants per group, potential participants were called, and a date for the focus group was set. Two trained research assistants fluent in the local Ga language facilitated the focus group discussions, which were held in both English and Ga.

The hypothetical case for the discussion was:

*“Nadine is 15 years old, presently in her third year in JHS (Junior High School) and lives with her parents in Kumasi. For over 6 weeks, she did not see her menses. She reported this to Johnson, her boyfriend. They went to the city pharmacy and bought a pregnancy test. It turns out that Nadine is pregnant (she has never been pregnant before).”*

General views regarding the perceived burden and reactions to adolescent pregnancies, predisposing factors, how they could be reduced, the decision-making process, and who had to take the final decision were explored. The principal investigator (LEB), research assistants from the University of Ghana who spoke the local Ga language and a youth mobilizer in Jamestown were involved in the focus group discussions.

Eight focus group discussions were carried out with a total of 54 participants in groups of 7. All participants reported being Christian (Table 1).

**Table 1:** Participant distribution

Target Group	Number of Participants (N)	Number of Focus Group Discussions (N)
Teachers	14	02
Parents	14	02
Adolescents (never been pregnant)	14	02
Teenage mothers	14	02
**Total**	**54**	**08**

### Data analysis

The interviews were recorded and transcribed into English and validated by an experienced qualitative researcher. The data were anonymized before analysis. After comparison and discussions, the researchers agreed on a coding frame in ATLAS.ti for Windows version 8. A thematic analysis approach was adopted for the data analysis, guided by the 5 nested hierarchical levels of the SEM (Fig. 1). Thematic analysis aims at identifying, analyzing and reporting themes (patterns) within data [19]. Elsewhere, its theoretical freedom provides an opportunity for an in depth exploration of complex and under researched phenomena like early adolescent pregnancy decision making and risk factors [19, 20]. We adopted the 6 phase thematic analysis approach proposed by Braun and Clarke in analyzing the data [19]. These phases are: familiarization with the data, generating initial codes, searching for themes, reviewing themes, defining and naming themes, and producing a report. The codes were applied to different blocks of the texts, and the transcripts were reviewed iteratively. Emerging themes were then discussed by two researchers. The initial coding process was open, and followed by an axial coding to establish meaningful connections among emerging themes.

## Results

### Perceived prevalence of adolescent pregnancy

All participants knew at least one adolescent around Nadine’s age (15 years old) or younger who had recently been pregnant. Our findings confirmed that early adolescent pregnancies (15 years of age or younger) were rampant, and somewhat normal. Yet our participants thought that it was a serious concern. The quotes below show how the respondents feel that this hypothetical case is a common phenomenon encountered by their community.

*“It is very common in the Ga communities. There are a lot in our neighborhood, and it is worrying and very sad” [Parent, F, 2]*

*“Girls of 13 and 14 years old give birth in my school, and their parents are the ones taking care of the babies” [Teacher, M, 3].*

*“I know someone who gave birth at the age of 15, and now at 34 she has five children. In our neighborhood, we have a lot of them who have given birth to several children at tender ages. When you are mature and have not given birth, you are insulted, even those of us who have one child are also insulted as barren” [Parent, M, 6].*

*“It is really shocking in this community. You see breastfeeding mothers, children you can never even imagine that they have started seeing their period. God should help this generation” [Parent, F, 1].*

### Perceived feelings after discovering the pregnancy

The perceived reactions of a pregnant adolescent after finding out that she is pregnant were almost always described as negative by the interviewees. These feelings ranged from “fear”, “anger”, “disappointment”, “frustration”, “miserable”, “regret”, “being shy”. The respondents thought that the adolescent in question would most likely experience a strong tension first between either keeping this information to herself or telling someone else. The overwhelming dilemma of what to do about the pregnancy (continue to birth or choose an abortion) was said to be the most challenging of the frustrations this adolescent has to deal with.

The feeling of frustration was often shared among the boys who get these adolescents pregnant, especially if they were young.

*“If the boy is say 15 or 16, he will be afraid and confused, as he cannot take care of the girl, let alone the child, too”[Parent, F, 11]*

### Why the high adolescent pregnancy rates?

Respondents were asked to brainstorm on the possible reasons for early adolescent pregnancy in Jamestown.

#### Lack of parental support

Respondents were unanimous that parents contributed immensely to the high prevalence of adolescent pregnancies in the community. It was mentioned repeatedly that discussing sex-related issues with children was considered a taboo in society. This complete lack of communication does not allow parents to get to know their children or perceive any changes, or allow the children to address their concerns to their parents. Most parents with their daily routines almost never had time to spend with their daughters. This negligence was exemplified by one parent, who mentioned that young girls could often go out at night and then come back without the parents even noticing. Parents’ failure to provide the basic financial needs of their children rendered the girls vulnerable to collecting money from boys and men, either to survive or to be liked by her peers.

*“Parents are to be blamed for all these unfortunate occurrences. A mother does not know how her daughter survives on a daily basis. When she (the mother) finishes eating, she goes to bed while these little girls will be taking money from boys, why would they not get pregnant?” [JHS 3].*

*“My friend noticed that her daughter was pregnant when she was already six months into her pregnancy. This means that she does not even have time to look at her own daughter” [Parent, M, 6].*

A parent was categorical about the fact that parents in the Ga communities (Jamestown) do not provide adequate care and attention to their children.

*“In Ga communities we don’t train our children. We as parents are equally guilty for not raising our children properly” [Parent, M, 9].*

*“I have the experience of what we are saying here near my home. This woman gave birth to six girls. Recently, the third girl got pregnant without knowing who was responsible for it. Parents are giving birth and reneging on their responsibilities of bringing up their children to be good adults” [Parent, F, 11].*

Sleeping with men or boys for financial reasons was a recurrent explanation of why many adolescents were getting pregnant. With low financial autonomy, adolescents were said to be more likely to succumb to peer pressure, to meet their financial needs by offering their bodies in exchange for money.

*“We have a lot of girls in such situations in the Ga communities. Most of them find it difficult to feed themselves and are put in such situations by irresponsible men” [JHS 8].*

*“We have many of those goats in this community. They are ready to sleep with girls, even younger than their grandchildren to satisfy their evil desires” [Teacher, M, 1].*

#### Reputation of the neighborhood

Respondents thought the neighborhood already carried a “bad” reputation, especially due to rampant adolescent pregnancies. Adolescents in this area were considered to be more promiscuous compared to other areas in Accra. Others reported that adolescents were generally known in this city to be disrespectful towards elders and parents.

*“Someone else will say he will never allow the child to ever live here because the teenagers here don’t live a good life” [Parent, M, 13]*

*“So I for instance will not allow my daughter to come and stay in this community” [Teacher, M, 3].*

Even outside the neighborhood, it was known that some employers refuse to offer jobs simply because of the bad reputation of the area. The activities and bars around the seashore were identified as a breeding ground for adolescent pregnancies. Most respondents thought that was where most men who buy sex for money recruit the adolescents.

*There’s a place at the shore called ‘One Conner’, come and see small girls and what they do at this place, it is an eyesore to Jamestown and its people.” [Parent, M, 9].*

#### Broken homes as risk for early adolescent pregnancy

Most respondents were of the opinion that broken homes were responsible for the poor education of the kids. Lack of proper education and advice predisposed the children to be easily influenced by negative peer pressure, and fail to abstain from sex. It was reported that some adolescents tend to do the same things they see their parents doing.

*If a child sees the mother always collecting money or sleeping with different men, she will think that is the right or normal way to live. Like mother, like daughter [Teacher, M, 9].*

*“I know of a twelve-year-old who gave birth. Her mother had a boyfriend and she would repeatedly send her to go ask for money from her boyfriend. As time went by, this girl succeeded in taking her mother’s boyfriend from her and got pregnant by this man; when the girl was asked who had made her pregnant, she said it was her mother’s boyfriend. Children from broken homes become misfits in society”[Teacher, F, 4].*

#### Knowledge and financial autonomy

Ignorance around sexuality issues was also raised as a possible explanation of high adolescent pregnancy rates in the area. Girls were thought not to have enough knowledge regarding the use of modern contraception and could be shy about buying them from the shops, for fear of being reported to their parents. Those who used sex for money were thought not to have any choices, especially when the men were not interested in using a condom. Some adolescents were reported as engaging in sex for money, in order to meet up with their friends.

*“The girls are poor, and their parents do not provide for them. They have no choice but to go in for these men” [Parent, M, I]*

*“They love to compete with their friends. Some will do anything to get money” [JHS 2]*

#### Technological factors

Exposure to social media, through use of the television and phones were considered important predisposing factors to adolescent pregnancies. Adolescents were thought to watch pornographic films due to a lack of parental control, which increased their passion for sex, and they practiced what they saw.

*“Parents do not spend time with their children; do not check what the children do with their phones” [Parent, F, 6]*

*“There is cheap pornography everywhere, with this internet thing and on the TV, children know everything. Since parents don’t care about what their children do, the children get used to these things, and before you know it, they simply practice what they see” [Parent, F,1]*

### Decision making

There was a general expression of perceived mixed feelings about keeping the baby or choosing an abortion.

#### Factors affecting the decision

The need for Nadine to pursue her education, her age, religious concerns, fears and awful stories of “victims” of induced abortions, fears of not being able to get pregnant in the future (secondary infertility), the readiness of the boy and family to take responsibility for the girl and the baby were issues that needed to be taken into consideration during the decision-making process. Most respondents were of the opinion that having an abortion in Nadine’s case was not an option, most especially on religious grounds. A few respondents, however, thought having an abortion was acceptable to allow Nadine to complete her studies. Continuing her education was an important consideration for some in the decision-making process. This should depend on whether the girl was intelligent or not.

The table below (Table 2) summarizes the main quotes deriving from the interviews regarding factors respondents thought needed to be considered in decision making involving the 15-year-old pregnant adolescent.

**Table 2:** Considerations in the decision-making process

Consideration	Supporting quotes
Need to pursue her studies	*She is too young. She needs an abortion to save her studies [JHS 13]*
Her age	*This is a child. She needs to get mature enough to be able to take proper care of the child [JHS3]*
Fear related to abortions (complications; death and secondary infertility)	*I know someone who also got pregnant and didn’t want to tell anyone, but later told her boyfriend and friends and was taken to Korlebu for abortion, unfortunately for her, she died the following day [Parent, M, 2]* *Abortion is not a good thing, and we have learnt our lessons from such experiences if because of abortion you are not able to give birth in future [Parent, M, 6]* *Iif she terminates it, she will die. So she has to just leave it and give birth [JHS7]* *She is too young. If she commits an abortion, who knows if she will be able to conceive again in the future? [JHS1]*
Partner and family responsibility	*“If Johnson’s parents can take responsibility for Nadine and the baby, she has to keep the pregnancy” [JHS3]* *“She will have to inform the man immediately. If the man agrees that he is responsible for the pregnancy, then she can give birth; if not, that is when abortion is considered.”[Parent, F, 11]*
Financial considerations	*Everything is basically about money. It is not easy to take care of a child, so only if there is money, then she should be allowed to give birth. [Teacher, M, 1]* *If the girl’s mother has money and is capable of taking care of the child, she can allow her to give birth so that she can take care of the child to enable her daughter to go back to school.[JHS3]* *Some parents are too stingy to use their money for abortion [JHS2]*
Religious considerations	*“Abortion is an unacceptable sin. For me, I was unable to complete school, but I now have a human being instead”[Parent, F, 1]* *“The Bible is telling us that children are from God so any conception must end up in childbirth; there is no need to decide what should be done with the pregnancy.”[Parent, M, 3]* *“A pregnancy should end in childbirth.* *She must not think of abortion because anything that is done with God ends well.”[Teacher, F,3]*

Parents generally thought there was no issue to be decided upon whatsoever. According to them, once pregnant, one has to give birth.

“*If you are a girl and you think you cannot take care of a child, then don’t have sex. Because having sex with a man could lead to pregnancy. If it happens, you simply have to give birth” [Parent, M, 3]*

Some parents would consider going in for abortions secretly to avoid the shame of their children having illegitimate children.

*“Some mothers keep the information from the public to avoid disgrace, especially when the child is intelligent, and then secretly abort the pregnancy for the child to enable her to go back to school” [Parent, M, 2].*

*If she is not doing well in school, then you can allow her to keep the pregnancy.*

Some very “religious” participants thought that abortions, under some circumstances, could be acceptable on religious grounds. With God being a God of mercy, He could easily forgive the young girl and the parents for taking such decisions, maybe to save the child’s education.

*Sometimes it is not as if you want to destroy the pregnancy but circumstances and the situation your daughter and yourself would go through may lead to such a decision. We pray to God to forgive us for taking such hard decisions and ask for His mercies to see us through successfully. God is a God of mercy [Parent, F, 4]*

Considering the fact that the adolescent would be in a dilemma either to opt for an abortion or continue the pregnancy to term, we sought to explore the respondents’ views regarding continuing until birth and having the child adopted later on. None of the respondents was in favor of adoption as an alternative to remaining pregnant or choosing an abortion. Some even thought that going in for adoption was worse than going in for an abortion.

*“Your mother is your mother. An adopted mother can never be like your mother” [Parent, M, 2]*

*“It is a horrible thing to deliver your own baby and give it away to another person to rise. How do you think that baby will feel?” [JHS 5].*

#### The final decision

In general, the respondents stated that the adolescent needs help in deciding on the outcome of the pregnancy. The persons to be involved included: parents (the girl’s, and at times, the parents of the partner), friends, or a trusted elderly person. Trusted family friends or elderly persons can be approached, especially considering the fact that the girl may be afraid to tell her parents. However, friends who would offer advice in favor of choosing an abortion were considered bad.

*She’ll first of all seek advice from her best friend. If her friend is a good one, she’ll advise her to let her parents know about her situation. On the other hand, if her friend is a bad one, she will advise her to abort it. [JHS 10]*

Others thought there was no decision to be taken in such circumstances. As long as she was pregnant, she simply needed support to carry on her pregnancy to term.

Regarding who should take the final decision in either keeping the baby or choosing an abortion, the overwhelming majority of the respondents recommended the father, followed by the mother. Others thought the partner should be the one to make the final decision. A minority of adolescents who had never been pregnant before reported that the girl was capable of making a final decision regarding her pregnancy outcome.

Table 3 summarizes the reasons why various actors should be the ones to decide on early adolescent pregnancy outcomes.

**Table 3:** Who should make the final decision and why?

Who makes the final decision?	Supporting quotes
The father	*The father has the final decision; He’s the only person who can say the final thing to either make me deliver or terminate it because he’s the family head [JHS 1,]* *The dad is the only person who has that authority to say I should give birth or terminate it because we have his name and besides, he’s the head of the family so before we do something, all of us ask his permission before we make the move [JHS 3]* *The girl’s father has the final say as to whether his daughter will abort the pregnancy or not. It is not the question of whether he cares for his daughter or not. The boy who impregnated the girl should not be involved at all because he has no right first of all to sleep with the girl to the point of making her pregnant [Parent 3].*
The mother	*Mothers have got a big role to play in the decision-making process. Mothers actually decide on the fate [Parent, M, 5]* *It is her mother who has the final say with her daughter’s pregnancy [JHS6]*
The two families	*I think the parents from both sides should come together to agree on how best they can solve the problem [Parent, F, 10].*
The girl herself	*Nadine knew that becoming pregnant could result from having sex, so she should know what to do with the pregnancy if she gets pregnant. [Teacher, M, 2]* *She is the only person who decides what to do with her pregnancy. She went and slept with Johnson and knew she could get pregnant. So she has to have decided beforehand what she would do if she gets pregnant [JHS 3].*
The partner	*Yes, I think that she should let her boyfriend know about it, because he is the one who impregnated her; if she doesn’t tell him, and the pregnancy is advanced before she tells him, he will deny the pregnancy [Teacher, M, 5].* *The one who has the final say is the man who impregnated the girl, whatever he says should be final. [Parent, F, 4]* *Nadine cannot decide for herself so she would have to decide with the boyfriend because he is responsible for the pregnancy. [Parent, M, 7]* *She needs her partner to sit by her to make the decision to abort it or keep it [Teacher, F, 6]*

### How can unintended pregnancy among “young” (15 years or less) adolescents be reduced in this community?

Participants believed that the introduction of sex education as early as possible would help raise awareness among adolescents regarding the consequences of unprotected sex.

*“We have to visit the various schools and gather such children within the age bracket of thirteen to fifteen and educate them about the dangers of sex to their education, lives, health, and the negative impact it brings to their future”[Teacher, F, 3]*

Most respondents thought that the parents were too closed to discuss sexuality issues with their children. Becoming more open was considered as empowering the girl’s knowledge regarding her status as a girl, and how to react when approached by men.

*“We can also gather them and listen to their problems and then educate them” [Parent, M, 2]*

*“We need to gather them in this community and educate them like we are doing here. This will help to reduce the the burden [Parent, F, 5]*

Others were in favor of policies to restrict the movement of adolescents at night. This should be coupled with stricter parental control.

***“***I think they should introduce the Rawlings era (former military leader and politician, who was president of Ghana between 1981 - 2001), where children under a certain age were arrested at night time. I was once arrested for coming home late from the market”* [Parent, M, 9]

Some parents thought that some adolescents might be possessed by a spirit of “stubbornness” which made them not heed their advice to abstain from having sex. God’s intervention through prayers was seen as the way to go in order to reduce adolescent pregnancies.

*First, we must instill in them the fear of God. Secondly, government must establish work for the youth from the ages of fifteen upward. Thirdly, we need soldiers in our communities to deal with girls who are seen late at night with men. [Parent, M, 9]*

*Only prayers because we have advised them like any other human being will do but it is not working. [Teacher, F, 3]*

*We need to pray and instill the fear of God in our children. It is only God who can change our situations here in Jamestown. The good people who were trained in this community are now doing well in life. [Teacher, M, 11]*

## Discussion

The aim of this study was to understand the perceptions of stakeholders regarding early adolescent pregnancy and decision making in Ga communities. We used a hypothetical story of a 15-year-old secondary school student who recently realized that she had not seen her menses for the past 6 weeks (Nadine) as a vignette. With all respondents knowing at least one or more adolescents who had been pregnant as early as 15 (in Nadine’s shoes) in the community, this translates into a presumably high prevalence of early adolescent pregnancies in Jamestown. The feeling of fear, shock, and disappointment reported by respondents is an indication that the young adolescent pregnancy is considered unintended in this case. Authoritarian parenting and the absence of an open communication climate between the parents and adolescents on sex related issues have been abundantly reported to predispose to adolescent pregnancy [11, 19–22]. Parents were also reported as spending very little time with their children, and almost never discussed any sex-related issues. Sex issues remain a taboo in parent-child communication within the Ga community. Kruger et al. reported similar findings in a qualitative study among 20 never pregnant adolescents in Bolgatanga, Ghana [11]. In their study, the adolescents and young women (less than 21 years of age) reported that they had never discussed any sex-related issues with their mothers. Comprehensive sex education was considered inadequate by our respondents. A predominance of predominance of abstinence-only messages within the school sex education packages have been reported from Ghana [23]. Adolescents who receive comprehensive sex education have a lower risk of pregnancy than adolescents who received abstinence-only or no sex education [21]. Comprehensive sexuality education is not only useful in preventing adolescent pregnancy rates, but could a long way in improving adolescent health in areas like denouncing rape attempts, sexual assault, and use improving uptake and use of modern contraception.

Religious reasons were not mentioned as protecting against, or an enabling factor for adolescent pregnancies. Sex is a taboo subject generally among Christians; this appears to be an expected finding since our sample was predominantly Christians [22, 24]. The Christian doctrine in most areas in Ghana stands by the abstinence-only doctrine, especially before marriage. In a case-control study among pregnant and non-pregnant adolescents in Ecuador, religiosity protected against adolescent pregnancy [25]. Further research elucidating how religiosity influences adolescent pregnancy is needed. In a study investigating adolescent decision making in Jamestown, religious considerations were not taking into consideration in the decision making process by the adolescents [12]. Insisting on the socio-economic advantages of staying longer in school, delaying marriage and childbirth at the community level could indirectly deter these young girls from getting pregnant in highly religious communities.

The lack of financial autonomy was a key risk factor for early adolescent pregnancy in our study. Providing sex for money has been identified elsewhere in Ghana as a main cause of adolescents engaging in sexual activity [11–23, 25]. In two qualitative studies in the Accra metropolis with teenage mothers, lack of money was a key factor that pushed adolescents to become sexually active [12, 24]. In economically disadvantaged conditions, and aggravated by the norm of male dominance, not only are the adolescents more prone to having sex earlier and more frequently, condoms could be underused, due to the financial dominance of men.

Exposure to the social media and television were considered as contributing factors to early adolescents’ pregnancy. They thought that the parents never spent enough time monitoring what the children watched or did with these media. Using these media to watch pornographic films was considered by some parents as encouraging the children to practice what they saw. In a qualitative study among out-of-school adolescents in a Muslim-dominated market in Nigeria, Odeyemi et al. found that pornography was associated with early initiation of sexual activity [26]. In a survey among 413 young Nigerian internet users, exposure to pornographic material was associated with increased engagement in having oral sex and having multiple sexual partners [27]. It is difficult to stop adolescents today from using the internet or watching television. Spending more time with the children and having open discussions with them regarding sexuality issues could be helpful. On the other hand, the Ghana health service has committed to making reproductive health education available online through an educational mobile app for Ghana Adolescent Health Program Service Providers (https://apkpure.co/ghs-adh-mapp/). Adolescents can easily access information regarding contraception, menstruation, pregnancy, puberty for girls, self breast examination and abstinence.

Table 4 summarizes the perceived risk factors for adolescent pregnancy in this community, explained using the SEM. Perceived risk factors touched all levels of the SEM. This suggests that interventions to improve upon the societal understanding of risk factors for adolescent pregnancies should seek a framework that effectively connects and contains this wide array of potential determinants.

**Table 4:** Perceived risk factors for unintended pregnancies seen through the lens of the socio-ecological model (SEM)

Perceived risk factors of early adolescent pregnancy	SEM levels
Lack of financial autonomy Inadequate knowledge of sexuality	**Individual (intrapersonal) level**
Lack of parent–adolescent communication on sexuality-related issues Inadequate amount of time spent with kids to understand their concerns Lack of parental control Single parenthood/broken homes	**Interpersonal level**
Sexuality-related issues seen as taboo in households Technological factors (misguided use of social media, pornography) Exclusion of sex education in the religious discourse	**Societal level**
Abstinence-only dogma dominant in school comprehensive sex-education curricula	**Institutional level**
Restriction of movement of adolescents to specific areas (beach bars) and during certain times of the day (late night movement) Monitoring of contents of radio and television channels	**Policy/enabling environment**

The factors considered before taking the final decision were personal (age, financial autonomy, need to continue schooling), interpersonal (partner responsibility and quality of parent-pregnant adolescent relationship), and societal (fear of dying during the abortion process, shame, religion, cultural acceptability as main head and decision-making authority in the family). At the institutional and policy levels, there were no available guidelines regarding adolescent pregnancy decision making within the reproductive health package of the Ghana Health Service. It is important to have a sound reflection upon this, as a healthy decision-making encounter with the health care provider allows for appropriate knowledge sharing and trust building. Distrust in the health care providers for fear of breach of privacy, as well as health care provider attitudes and religious beliefs regarding safe abortion services have been reported as negatively influencing the access to and use of reproductive health services in Ghana [12, 24, 28].

Adolescents coming from single-parent homes were considered to be at greater risk of an early adolescent pregnancy, compared to those whose parents were in stable marriages [29]. These findings are consistent with reports from Ecuador, where pregnant adolescents were more likely to be from divorced families compared to their non-pregnant counterparts [25]. This could be explained by the fact that those parents may work harder, spend less time with their children, or may have generally lower earnings available to take care of the needs of their children. In contrast, in a quantitative exploratory study among 121 South African adolescents and young adults using a parenting authority questionnaire from the West Coast of South Africa, single parenting and divorced parental status were not associated with adolescent pregnancy [30].

The overall perception of the neighborhood as “bad” can be viewed through the lens of the social disorganization theory [31]. This theory is an example of an ecological framework and posits that crime is not randomly distributed, but occurs more frequently in ‘bad’ neighbourhoods than in ‘good’ neighbourhoods. Neighborhood characteristics (the schools, perceptions about schooling, access to schools, beach, and clubs in the case of Jamestown) are of the utmost importance in the elaboration and implementation of sexual and reproductive health interventions.

As reported by respondents, becoming adulthood without having a child attracts labels of being infertile for women. In our study, proof of fertility was not frequently mentioned as a possible explanation for Nadine’s pregnancy. This could be explained by the fact that the vignette was a student and generally expected to continue her studies. Having babies as early as possible in the Ga communities is also culturally desirable [3, 12]. This suggests that sending girls to school could counteract the early adolescent motherhood desirability in the community.

Going in for an abortion was considered to be justifiable for girls who were intelligent in school. In a questionnaire-based study examining how adolescents intend to resolve a pre-marital pregnancy among never married, sexually active adolescents in South Central Indiana, career-oriented participants reported more positive attitudes towards abortions [32]. The diversity in experiences (from fear of death, secondary infertility, to bleeding) reported by respondents could be an indication of unsafe abortion practices going on in this community. Almost half (45%) of abortions recorded in Ghana are unsafe [21]. Accepting or denying taking responsibility for the pregnancy has been reported to have a direct relationship with the final decision [23, 33]. Others thought if she could get pregnant, she should be able to decide on what to do with her pregnancy. These assertions could mean these participants think the adolescent can take informed (autonomous) decisions. Using vignette-based focus group discussions has been reported to be less personal, and allows respondents to be open regarding sensitive issues [17, 18]. In this study, the father was overwhelmingly considered to be the one to take the final decision regarding the pregnancy outcome. In an earlier report [12] concentrating on the experiences in decision making among adolescents who had continued their pregnancy to term (adolescent mothers) and those who had had at least one abortion before, almost all adolescents (28/30) reported taking the final decision either to keep the pregnancy or to terminate it on their own. Perceived and experienced decision making differ in this sociocultural context. This is an important consideration for policy makers when designing adolescent friendly reproductive health services.

### Limitations

The influence of religion on perceived decision-making considerations cannot be representative of the community as our study was exploratory. The smaller group of the Muslims and traditionalist views were not explored in this study, as all participants were Christians. Partners have been reported to play significant roles in the decision-making process. In this study, it was impossible to ascertain the role and aspirations of the partner. The health of the relationship with the partner could have an influence on the adolescent decision, which was not explored in this study. Adolescents who feel very close to their partners have been reported elsewhere as being less disposed to opt for a pregnancy termination [28, 32]. Further studies should consider the roles of health care providers, as they are also important in the decision-making and care processes.

## Conclusion

Early adolescent pregnancies were considered frequent in this area. To reduce adolescent pregnancy rates in this community would require interventions that target individual, interpersonal, societal, and institutional factors. Inadequate parental support and open communication regarding sexuality issues, poverty, dominance of abstinence as the only dogma in sex-education packages in school, and the growing use of television and social media were considered to be the major predisposing factors for high adolescent pregnancy rates. Culturally sensitive strategies are required to carefully introduce adequate and age-specific comprehensive sex-education packages in households and schools and among religious leaders. The final decision to choose an abortion or to continue the pregnancy to term was predominantly considered to be left in the hands of the adolescent’s father. Encouraging parents to be more open to discussing issues regarding adolescent sexuality could be very useful in improving the adolescent’s reproductive health.

## Data Availability

All data is available upon request.

## Abbreviations

SEM: Socioecological model
CDC: Centre for Disease Control
FDG: Focus group discussion
